# Tonal Auditory Discrimination Training in Youths: Design, Implementation, and Evaluation of Game-Based Versus Non–Game-Based Systems in a Cross-Sectional Study

**DOI:** 10.2196/92496

**Published:** 2026-06-22

**Authors:** Sergio M-Cam, Daniela Acosta, Karla I Cruz Ornelas, Priscila Montoya Beltrán, Luz M Alonso Valerdi, David I Ibarra Zarate

**Affiliations:** 1 Escuela de Ingeniería y Ciencias, Tecnológico de Monterrey, Ave Eugenio Garza Sada 2501, Monterrey, NL, 64849, Mexico, 52 (81) 8358-2000

**Keywords:** game-based training, gamification, auditory discrimination, auditory rehabilitation, user experience, usability

## Abstract

**Background:**

Auditory discrimination training is widely used to supplement aural habilitation and rehabilitation in individuals with hearing or auditory challenges. Recently, gamification has been introduced as a strategy to enhance attention, motivation, and engagement during training. However, the relationship between user experience (UX) and auditory discrimination performance remains unclear. In addition, maintaining long-term interest in auditory training is still a challenge, particularly in younger populations, highlighting the need for more engaging and user-centered approaches.

**Objective:**

The objective of our study was to develop and compare 2 pure-tone auditory discrimination training systems: a game-based system with dual-task gamified activities and a non–game-based control system with identical auditory tasks but without gamified elements. We further aimed to evaluate differences in UX, engagement, usability, and behavioral auditory discrimination performance between the two systems.

**Methods:**

A 3-stage process (design, implementation, and evaluation) yielded beta versions of both systems. Both platforms were developed using identical auditory stimuli, task logic, and performance metrics to isolate the effect of gamification, differing only in the interface and game elements. In the evaluation stage, a cross-sectional study with 11 young adults (aged 18-30 y) was conducted, where participants completed usability, UX, and engagement questionnaires after using each system. Behavioral performance was assessed through mean response time, proportion of correct responses, the Balanced Integration Score, and a novel Auditory Discrimination Performance Index integrating the Weber fraction and response time. UX was measured using the User Experience Questionnaire, the Post-Study System Usability Questionnaire, and the User Engagement Scale-Short Form. Statistical analyses included Shapiro-Wilk tests, paired *t* tests, and Wilcoxon signed rank tests.

**Results:**

The game-based system produced significantly higher scores in 6 of 11 evaluated questionnaire dimensions, including focused attention, aesthetic appeal, reward, attractiveness, stimulation, and novelty, while no significant differences were found in most auditory discrimination performance metrics. Specifically, measures such as response time, accuracy, and Balanced Integration Score showed comparable results between systems. Usability-related outcomes showed a slight advantage for the non–game-based system, whereas all engagement-related domains consistently favored the game-based system, indicating improved user involvement and overall engagement without compromising performance.

**Conclusions:**

These findings suggest that gamification can substantially improve UX and engagement without degrading short-term discrimination performance. This indicates that gamified auditory training systems may enhance user adherence without compromising task effectiveness. This study is innovative in that it directly compares game-based and non–game-based auditory training systems under controlled conditions, isolating the effect of gamification. Unlike other studies that primarily evaluate gamified systems without a control condition, this work clarifies the specific contribution of game elements to UX and performance. These findings contribute to the development of more effective, user-centered auditory training technologies and highlight the need for future longitudinal studies to determine whether these advantages translate into sustained auditory benefits across tasks.

## Introduction

Aural habilitation and rehabilitation support individuals in adapting to challenging hearing situations such as the use of hearing aids [1], bone-conduction implants [2], or cochlear implants [3]. These approaches are also relevant for populations requiring improved auditory performance, such as those with auditory processing disorders, including difficulties in localization, lateralization, auditory discrimination, pattern recognition, and temporal processing. Adaptation at early stages can be a complex task, as individuals must learn to process whole sound domains in the best way [4]. Main difficulties include accurately identifying and discriminating speech from environmental sounds (including noise), locating sound sources, and particularly listening to music [5,6], which can be quite difficult. This adaptation process may extend over months to years, since it relies not only on sensory changes but also on neural plasticity [7]. Recommended strategies for this population include auditory-verbal therapy, mainly in children [8], and auditory training [9], which contain core exercises that enhance perceptual refinement [10]. As these activities extend over long aural rehabilitation periods, individuals often engage in lengthy sessions that must be integrated into their daily routines [8]. In this way, user-centered and engaging approaches may have better reception among young people and children [11], enhancing motivation and interest in longitudinal activities [12].

In recent decades, a series of systems and tools have been developed to improve accessibility and personalization [1,13], enhancing the population’s attention and rehabilitation performance. New technologies and approaches have been followed by a user-centered design and have been validated through user experience (UX) tests [14]. Yet not all of them have followed processes of user-centered design and UX evaluation that support engagement in attractive and modern gameplay and genres, fitting auditory discrimination tasks and ensuring usability—critical elements for achieving game goals. Thus, studying the real impact and analyzing the efficiency of gamification in auditory discrimination training could be quite complex [15], especially when identifying key improvement elements when no control groups are considered.

Recent studies have evaluated game-based auditory training through questionnaires assessing usability, gameplay, and attractiveness [16,17], building design and evaluation methodologies ranging from game-user research to beta testing and assessment. Other recent studies have compared gamified auditory training performance with control groups performing different tasks [18]. However, very few studies to date have compared game-based auditory training with equivalent non–game-based auditory discrimination activities to measure both differences in general UX (focused on usability and engagement) and behavioral performance [19,20].

A key consideration of auditory training is the selected stimuli. Most speech-based training tools are mainly available in English, which makes accessibility difficult for youths and children whose first language is not English. As an alternative, some studies have suggested a transfer effect of other auditory stimuli that may improve complex auditory perception, such as speech [21,22]. Some analyses about the effects of nonverbal training, such as music and other types of auditory temporal training, have demonstrated improvement in language skills [23], while some others have reported no differences [24]. Still, this approach addresses the challenges of language-dependent systems development.

Addressing these gaps is crucial for developing inclusive, evidence-based tools that support diverse populations and, importantly, link enhanced UX to long-term performance improvements [25]. The aim of this study was to develop and directly compare 2 pure-tone auditory discrimination training systems—a game-based system and a non–game-based system—while controlling for auditory stimuli and task structure. Specifically, this study sought to evaluate differences in UX, engagement, usability, and auditory discrimination performance between both approaches. We hypothesized that the game-based system would improve engagement and UX without negatively affecting auditory discrimination performance compared to the non–game-based system. In this manner, this work makes 3 main contributions. First, it presents the design and beta evaluation of 2 matched auditory discrimination systems that isolate the effect of gamification by keeping auditory stimuli and task logic identical. Second, it introduces an Auditory Discrimination Performance Index (ADPI) that combines just-noticeable differences (JNDs) and reaction times into a single performance metric. Third, it provides empirical evidence on how specific gamification elements influence UX, engagement, and behavioral performance in pure-tone training tasks.

## Methods

### Development Stages

**Figure 1.**
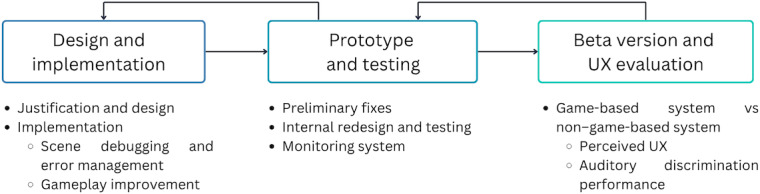
Overview of the methodology for design, implementation, testing, and evaluation of both game-based and non–game-based auditory training systems. UX: user experience.

The methodology for both systems’ design, implementation, and evaluation was adapted from [16,19] and consisted of (1) problem definition and initial design, (2) creation of a prototype and testing, and (3) UX, usability, and engagement evaluation of the beta version, as described in Figure 1.

### Systems Design and Implementation

#### Auditory Discrimination Tasks

**Figure 2.**
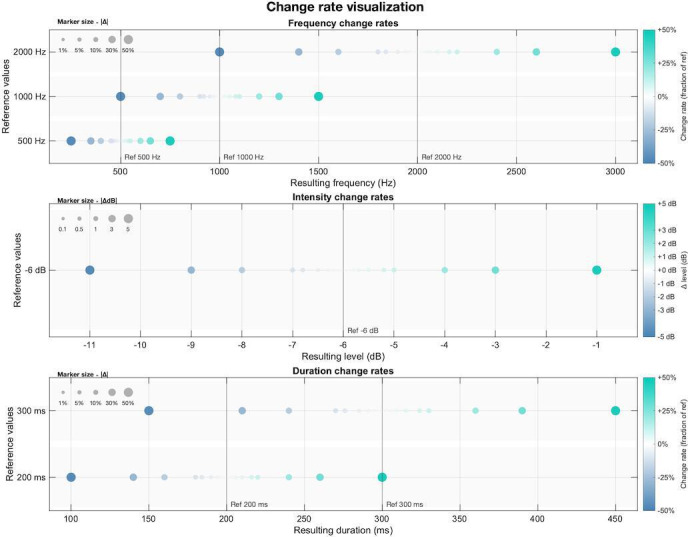
Pitch, duration, and frequency references and targets. Top: pure-tone pitch references of 0.5, 1, and 2 kHz and targets from 1% to 50% change rates. Middle: pure-tone intensity reference of −6 dB FS and targets from 0.1 to 5 dB changes. Bottom: pure-tone duration references of 200 and 300 ms and targets from 1% to 50% change rates. Reference tones mainly have a pitch of 0.5 kHz, an intensity of −6 dB FS, a duration of 200 ms, and an envelope with fade-in and fade-out of 5 ms.

Two different auditory training systems were developed: a game-based training system (GBS) and a non–game-based training system (NGBS). Each of them delivers sound stimuli for auditory discrimination of pitch, duration, and intensity, as the basis of the auditory discrimination hierarchy proposed in Sindrey [26]. Pure-tone pitch and intensity references and targets were selected based on the main speech features across languages [27], as shown in Figure 2. Duration references and targets were selected based on consonant-vowel timing [28], as shown in Figure 2. In the GBS, however, these tasks are presented in a gamified format based on an infinite runner genre (Licensed Infinite Runner Engine in Unity), as used by other similar game-based training systems [16,22], while the NGBS offers the same pure-tone auditory discrimination tasks in a nongame platform. To guarantee comparability, both systems used identical auditory stimuli, task logic, and performance metrics. Only the interface and gamification elements differed.

#### Non–Game-Based Training Platform

The NGBS delivered the same auditory stimuli and discrimination tasks using a static, minimalist graphical interface. Visual feedback was restricted to the participant’s response to each individual trial, with no indication of score, animation, time pressure, or level of engagement. This methodology required participants to focus only on sequences of pure-tone discrimination tasks, providing a low-engagement control condition.

#### Game-Based Training Platform

During GBS training, participants played in an infinite-runner format in which they guided an animated character through an obstacle course as they completed different auditory discrimination tasks. This dual-task structure was intended to increase cognitive load and promote sustained engagement by combining behavioral training with interactive gameplay, following a classical dual-task paradigm [25], where the primary task is a pure-tone discrimination task and the secondary task is the infinite-runner genre game. Correct responses on the primary discrimination task allowed them to continue playing and triggered positive audiovisual feedback, whereas errors reduced lives and elicited corrective audiovisual feedback. Correct responses on the secondary task allowed them to continue increasing their score but did not directly affect the primary task’s performance metrics.

#### Implementation Process

Both platforms were implemented in Unity (C#) using the FMOD audio engine and following an iterative, multistage development cycle.

##### Prototype and Alpha Phase

The design and genre were selected based on popular infinite-runner mobile games and previous implementations in auditory tasks [16,22]. Core mechanics (jumping and maneuvering) were selected based on their connection with the main discrimination goal, as described in [29], and were implemented and internally tested to ensure timing precision and data integrity. Enhancements included improved instructions, sound-level adjustment, and basic visual feedback.

##### Beta Phase

Dynamic scoring and refined user interface elements were introduced. Feedback from pilot testing guided the correction of perceptual calibration issues, menu structure, and visual clarity. The stable versions used for validation and experimentation incorporated all refinements and enabled the automated export of performance metrics.

A comparison of platform features is presented in Table 1, summarizing differences in task presentation, feedback, scoring, and data logging between the GBS and NGBS. This table highlights how gamification elements were deliberately implemented in the GBS and omitted from the NGBS to isolate the effect of game mechanics. A more detailed game design sheet is shown in Table S1 (Multimedia Appendix 1).

**Figure 3.**
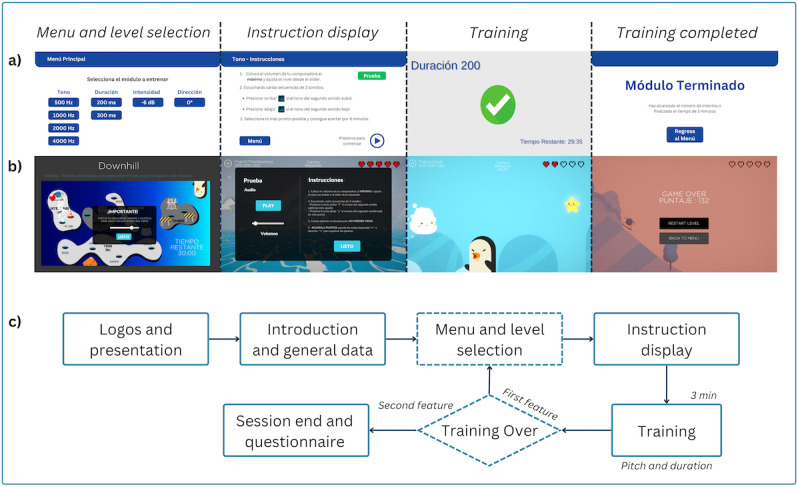
Systems interfaces and user flow. (A) Non–game-based system and (B) game-based system interfaces of 4 stages (menu and level selection, instructions display, training, and training over). (C) User flow that participants followed when testing each system for both sound features (duration and pitch).

A version of each auditory discrimination system was exported for Windows OS for controlled testing. Each version, as well as the system user flow, is shown in Figure 3.

**Table 1.** Comparison of game-based and non–game-based systems features.

Feature	Non–game-based system	Game-based system
Task presentation	Static auditory stimuli	Embedded within a dynamic obstacle-navigation game
Feedback	Text-based success or error	Animated success or error, points, lives, and rewards
Engagement cues	Minimal	Visual animations, background music, rewards, and challenges
Data logging	Responses, intervals, training time	Responses, intervals, scores, lives, and session data
Questionnaire integration	Pop-up reminders	Integrated pop-ups within the game flow
Instructions	Text instructions	Text and interactive in-game guidance

### Systems Testing and Evaluation

#### Stage 1: Prototype Testing

The first validation stage focused on technical reliability and preliminary user feedback. Six adult volunteers completed 5- to 10-minute sessions with each platform. Participants performed sample tasks, reported interface issues, and provided qualitative feedback on clarity, responsiveness, and sound calibration. Observations from this stage informed adjustments to auditory cue presentation, error handling, and menu navigation.

#### Stage 2: Beta Testing

The goal of the second stage was to evaluate usability, engagement, and performance in the near-final versions. A total of 11 participants completed structured training blocks on both platforms in counterbalanced order. After each session, they completed 3 standardized questionnaires: (1) the User Experience Questionnaire (UEQ), (2) the Post-Study System Usability Questionnaire (PSSUQ), and (3) the User Engagement Scale-Short Form (UES-SF). Feedback guided final refinements, including clearer instruction prompts, improved sound-level calibration, and enhanced data-logging reliability.

The current work focuses on findings from the beta testing stage prior to the full-scale deployment of the final version.

### Ethical Considerations

This study was conducted in accordance with the principles outlined in the Declaration of Helsinki. Ethical approval was obtained from the Ethical Committee of the Instituto Tecnológico y de Estudios Superiores de Monterrey Engineering School with ID EHE-2023‐11 and was registered in the International Standard Randomized Controlled Trial Number registry (ISRCTN43661994).

Participants were given informed consent prior to study enrollment. The consent stated the procedure and described the materials, ensuring voluntary participation. Participants’ data were anonymized prior to analysis to protect participants’ privacy and confidentiality. No identification of individual participants or users is included in this study. No monetary compensation was given to the participants.

### Participants

#### Sample Size, Power, and Precision

The sample for the beta testing consisted of 11 participants. The sample size was determined based on usability testing guidelines. In general, a cohort between 5 and 10 participants is often considered sufficient to identify major usability problems [19,30]. The achieved sample size met these recommendations and corresponded to the total number of participants available during the study period.

#### Sampling Procedures and Participant Characteristics

Participants were recruited via online flyers and personal invitations through a voluntary response sampling process from the beginning of September to the end of October 2024. All participants were adults aged 18 to 30 years (mean 21.82, SD 3.13 y) and were enrolled in either a bachelor’s or master’s program at Tecnológico de Monterrey. The sample consisted of 11 participants with a male-to-female ratio of 1.75:1. All participants recruited participated in the study.

Sessions were done at the NeuroTechs laboratory within Tecnológico de Monterrey. At the beginning of the session, participants were informed about the overall purpose, procedure, and relevance of the study. Following written informed consent, participants completed an initial questionnaire comprising demographic data and questions aimed at evaluating their overall health condition and gaming hours per week.

#### Inclusion and Exclusion Criteria

Inclusion criteria required the participants to be aged between 18 and 30 years, report normal hearing abilities, and have no diagnosis of neurological, auditory, or attentional disorders. In addition, no participant was undergoing any medical treatment at the time of the experimental procedure. If any of these requirements were not met, participants would not be enrolled in the study.

### Study Design and Procedure

#### Conditions and Design

This study followed a within-subjects cross-sectional experimental design in which participants interacted with both systems. The order of exposure to the systems (NGBS or GBS) was randomized for each participant to avoid potential order effects. This corresponds to an experimental manipulation with a randomized condition order.

#### Instrumentation

A Class-1 sound level meter (B&K Type 2270) was set to register the background noise levels during the use of each system to ensure that the experimental phase was conducted in a quiet environment and that noise was not a relevant factor affecting performance. Participants were seated comfortably in front of a laptop with the required software installed. Steren AUD 230-NE headphones were provided to ensure uniform quality of the delivered audio across all sessions with participants.

#### Procedure and Data Collection

Participants were asked, via a written and established set of instructions, to proceed with the interaction with the first system (randomly assigned). Participants were required to navigate through the *Logos*, *Introduction*, and *Level Selection* scenes before starting the first task to ensure the graphical user interface was understandable.

Once the first feature was selected (pitch or duration), instructions were displayed with the auditory discrimination tasks they would perform. Participants had to listen to continuous auditory sequences of 2 sounds, following a cue-target paradigm with a 2-alternative forced-choice response, and determine whether the target sound had increased (pressing the up button) or decreased (pressing the down button) compared to the cue in the sound feature that was being tested (pitch or duration). Subsequently, they could select the following sound feature (pitch or duration) in the same way. Auditory discrimination tasks were run for 3 minutes per feature. Upon completion, participants completed the UEQ, the PSSUQ, the UES-SF, and the Auditory Discrimination Performance Test for Discrimination Tasks.

**Figure 4.**
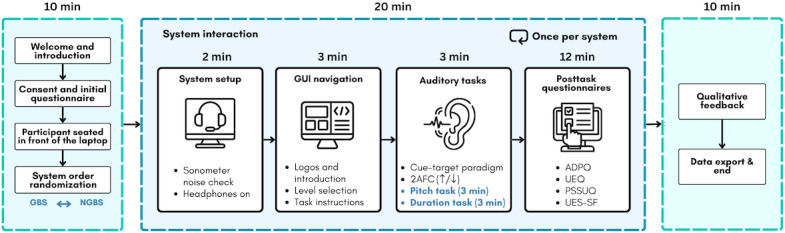
Overview of the experimental procedure for both systems. Each system (randomly assigned) received its own interaction phase with behavioral performance and user experience assessment. 2AFC: 2-alternative forced-choice; APDQ: Auditory Discrimination Performance Questionnaire; GBS: game-based training system; GUI: graphical user interface; NGBS: non–game-based training system; PSSUQ: Post-Study System Usability Questionnaire; UEQ: User Experience Questionnaire; UES-SF: User Engagement Scale-Short Form.

The procedure was then repeated for the second system, from setting up the sonometer to the completion of the questionnaires mentioned above. All data generated throughout the session were automatically saved and exported to a compressed ZIP file available for download from the systems for further analysis. An overview of the session and experimental procedure times is illustrated in Figure 4.

### Measures and Covariates

#### Balanced Integration Score

The Balanced Integration Score (BIS) is a measure that combines speed and accuracy from task responses while attenuating the speed-accuracy trade-off by giving equal weights to both components [31]. BIS is defined as Equation 1.


(1) $$\text{BIS}=z_{\text{PC}}-z_{\text{MRT}}$$

where *z*_PC_ and *z*_MRT_ correspond to the standardized values (*z* scores) across the observed proportion of correct answers (PC) and mean reaction time (MRT) in seconds from all subjects and conditions.

#### Auditory Discrimination Performance Index

The ADPI is a proposed integrated version of the inverse efficiency score [32] for auditory discrimination tasks, considering the Weber fraction *K* obtained by dividing the JND by the reference value of the corresponding attempt, and the MRT of JND, defined as the average time in seconds that each participant took to correctly answer the attempts made only at the selected JND. JND and the Weber fraction have been used to measure discrimination thresholds in challenging hearing conditions [33] or increased cognitive load tasks [34]. ADPI was computed as shown in Equation 2.


(2) $$\text{ADPI}=\frac{{\text{MRT}}_{\text{JND}}}{K\times 100}$$

A JND was considered valid only if the participant achieved at least 75% accuracy for that specific stimulus. If the threshold was not yielded, the next smallest JND was evaluated until a valid one was identified. For instance, suppose a participant selected the pitch modality with a reference value of 500 Hz and was tested with pitch differences of 5, 10, and 20 Hz relative to the reference value. If they did not achieve 75% accuracy at 5 Hz, but they did at 10 Hz, then the valid JND would be set at 10 Hz. Subsequently, the corresponding *K* value would be 10/500=0.02. In addition, if the participant correctly answered 4 times for that JND stimulus, the mean response time of those 4 attempts is the MRT of the JND.

#### Questionnaires

To compare user engagement, usability, and experiential perceptions between systems, 3 validated questionnaires were presented to participants: UEQ, PSSUQ, and UES-SF. UEQ evaluates attractiveness, perspicuity, efficiency, dependability, stimulation, and novelty on a differential scale of 26 bipolar adjective pairs rated on a 7-point Likert scale transformed into a numerical scale of −3 to +3 [35]. PSSUQ assesses system usefulness, information quality, and interface quality through a 1 to 7 scale, where lower scores indicate better usability [36]. UES-SF measures focused attention, perceived usability, aesthetic appeal, and reward on a 1 to 7 Likert scale, where higher scores indicate stronger engagement [37].

#### Covariates

No covariates were included in the analyses.

### Quality of Measurements and Data Diagnostics

Measurement quality was supported using a controlled laboratory environment, standardized hardware (headphones and computer setup), and consistent task instructions across participants. All sessions and procedures were supervised from informed consent until the last task was completed by each participant. No missing data were reported. Prior to performing statistical tests, data were first inspected for normality (Shapiro-Wilk tests) to select an appropriate analysis strategy.

### Analytic Strategy

Analyses were conducted to evaluate differences between systems across behavioral and questionnaire-based outcomes. For the in-game key performance indicators (KPIs), Python 3.12.12 was used to perform all statistical computations with the SciPy Stats library. The tests were carried out by comparing NGBS and GBS conditions paired by training feature (pitch or duration). Data were first inspected for normality using Shapiro-Wilk tests. The parametric paired *t* test was used when normality conditions were met. Otherwise, the nonparametric alternative, Wilcoxon signed rank test, was applied. Statistical significance was set at *P*<.05 for all tests conducted.

For questionnaire assessment, the Wilcoxon signed rank test using MATLAB 2025a and the Statistics and Machine Learning Toolbox was used to determine statistically significant differences between both systems across all questionnaire dimensions, considering the ordinal nature of the data. Mean scores, SD, *P* values, and effect sizes (*r*) values were calculated for each dimension per user, per questionnaire, and per system.

## Results

### Preliminary Data

**Figure 5.**
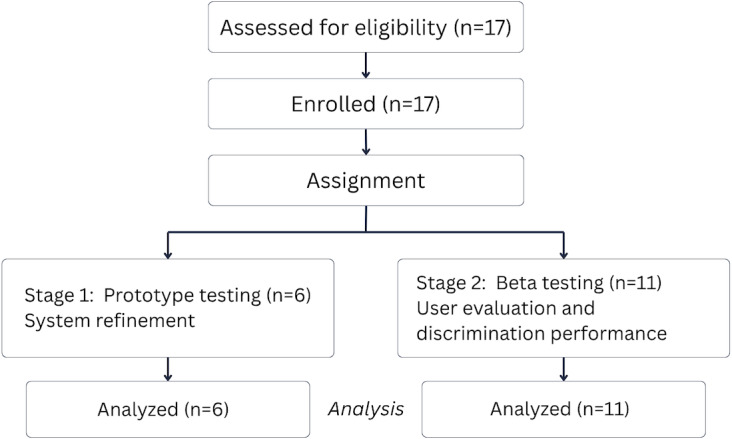
Flow of participants through each stage describing enrollment, assignment, and analysis samples.

All participants reported no hearing or visual impairment, no attentional or neurological disorders, and no current use of medication at the time of the experiment. As general information for the study, participants self-reported information regarding their sleep, eating, and hydration habits, as well as their average weekly time spent in hours playing video games. A participant flow diagram adapted from JARS guidelines is presented in Figure 5.

Regarding gaming exposure, 9 participants reported playing video games less than 4 hours per week, 1 participant reported between 4 and 8 hours, and 1 participant reported between 8 and 12 hours. This variable was included as a proxy for prior familiarity with interactive and gamified environments, which can influence both usability and engagement outcomes.

Due to limited variability in gaming exposure, no clear exploratory relationship was found between demographics and average weekly hours spent playing video games.

### Questionnaire Outcomes

**Figure 6.**
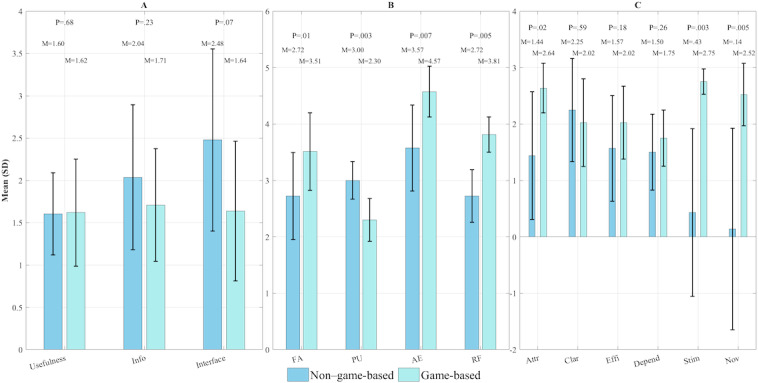
Questionnaires’ results for both systems. (A) Bar graphs showing mean, SD, and *P* value results in the Post-Study System Usability Questionnaire per dimension. (B) Bar graphs showing mean, SD, and *P* value results in the User Engagement Scale Short-Form per dimension. (C) Bar graphs showing mean, SD, and *P* value results in the User Experience Questionnaire per dimension. AE: aesthetic appeal; Attr: attractiveness; Clar: clarity; Depend: dependability; Effi: efficiency; FA: focused attention; Nov: novelty; PU: perceived usability; RF: reward factor; Stim: stimulation.

For all 3 questionnaires, participants recorded their answers across every dimension and system. For comparison between the GBS and NGBS, bar plots were generated to represent results and highlight differences in UX and overall performance patterns, as shown in Figure 6. Questionnaire responses showed significant differences and greater results in focused attention, aesthetic appeal, reward factor, attractiveness, stimulation, and novelty domains when using the gamified version.

Although the PSSUQ domains and the perceived usability domain of the UES-SF scored slightly higher in the NGBS than in the GBS, this resulted in a significant difference in only 1 of the 4 domains.

Afterward, a comprehensive statistical analysis was performed, which included the calculation of metrics such as mean score, SD, *P* value, and *r* coefficient, as presented in Tables 2-4.

**Table 2.** Statistical results of Post-Study System Usability Questionnaire domain for both systems.

Statistic	Dimensions					
	Usefulness^a^		Info^b^		Interface^c^	
	GBS^d^	NGBS^e^	GBS	NGBS	GBS	NGBS
Mean (SD)	1.619 (0.63)	1.60 (0.48)	1.70 (0.66)	2.03 (0.85)	1.63 (0.82)	2.47 (1.07)
95% CI	1.2 to 2.04	1.28 to 1.92	1.26 to 2.14	1.46 to 2.6	1.08 to 2.18	1.75 to 3.19

a *P*=.68; *r*=0.02.

b *P*=.22; *r*=−0.32.

c *P*=.07; *r*=−0.76.

d GBS: game-based training system.

e NGBS: non–game-based training system.

**Table 3.** Statistical results of User Engagement Scale-Short Form questionnaire domain for both systems. Significance was defined as *P*<.05 and indicated with an asterisk.

Statistic	Dimensions							
	Focused attention^a^		Perceived usability^b^		Aesthetic appeal^c^		Reward^d^	
	GBS^e^	NGBS^f^	GBS	NGBS	GBS	NGBS	GBS	NGBS
Mean (SD)	3.51 (0.68)	2.72 (0.77)	2.29 (0.37)	2.99 (0.33)	4.57 (0.45)	3.57 (0.76)	3.81 (0.31)	2.72 (0.46)
95% CI	3.05 to 3.97	2.2 to 3.24	2.04 to 2.54	2.77 to 3.21	4.27 to 4.87	3.06 to 4.08	3.6 to 4.02	2.41 to 3.03

a *P*=.01*; *r*=1.12.

b *P*=.003*; *r*=−1.7.

c *P*=.007*; *r*=1.26.

d *P*=.005*; *r*=2.01.

e GBS: game-based training system.

f NGBS: non–game-based training system.

**Table 4.** Statistical results of User Experience Questionnaire domain for both systems. Significance was defined as *P*<.05 and indicated with an asterisk.

Statistic	Dimensions											
	Attractiveness^a^		Clarity^b^		Efficiency^c^		Dependability^d^		Stimulation^e^		Novelty^f^	
	GBS^g^	NGBS^h^	GBS	NGBS	GBS	NGBS	GBS	NGBS	GBS	NGBS	GBS	NGBS
Mean (SD)	2.63 (0.43)	1.43 (1.13)	2.02 (0.77)	2.25 (0.91)	2.02 (0.64)	1.56 (0.93)	1.75 (0.5)	1.5 (0.67)	2.75 (0.22)	0.43 (1.48)	2.52 (0.55)	0.13 (1.78)
95% CI	2.34 to 2.92	0.67 to 2.19	1.5 to 2.54	1.64 to 2.86	1.59 to 2.45	0.94 to 2.18	1.41 to 2.09	1.05 to 1.95	2.6 to 2.9	−0.56 to 1.42	2.15 to 2.89	−1.07 to 1.33

a *P*=.02*; *r*=0.95.

b *P*=.59; *r*=−0.18.

c *P*=.18; *r*=0.45.

d *P*=.26; *r*=0.36.

e *P*=.003*; *r*=1.66.

f *P*=.005*; *r*=1.44.

g GBS: game-based training system.

h NGBS: non–game-based training system.

### In-Game KPIs

During auditory discrimination tasks, background noise was recorded when both systems were used. Background noise for the GBS had a mean equivalent continuous A-weighted sound pressure level of 43.21 (SD 3.39), and for the NGBS, it had a mean equivalent continuous A-weighted sound pressure level of 37.79 (SD 2.08), which means that for both systems, the background noise was very low during training and may not affect discrimination performance.

#### Balanced Integration Score

**Figure 7.**
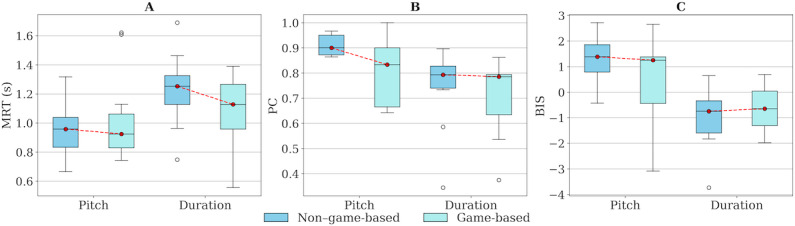
Performance results for both systems. (A) Boxplots of mean reaction time (MRT) by system and training feature. (B) Boxplots of proportion of correct answers (PC) by system and training feature. (C) Boxplots of Balanced Integration Score (BIS) by system and training feature.

Figure 7 presents the results of MRT, PC, and BIS grouped by system and training feature. To facilitate visual comparisons across systems, a red dotted line connects the medians corresponding to the same training feature.

As can be seen for MRT and PC, the GBS medians tend to be slightly lower than those of the NGBS. However, this is not clearly observed for BIS.

#### Auditory Discrimination Performance Index

**Figure 8.**
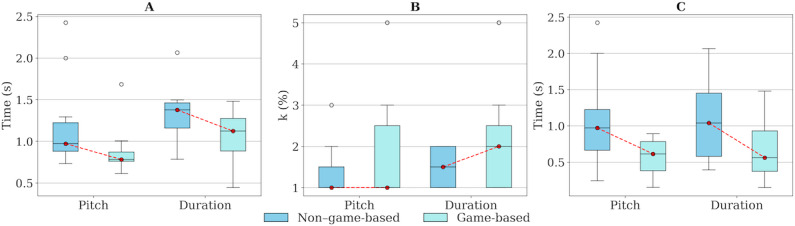
Performance results for both systems. (A) Boxplots of mean reaction time (MRT) by system and training feature. (B) Boxplots of Weber fraction (*K*) by system and training feature. (C) Boxplots of Auditory Discrimination Performance Index (ADPI) grouped by system and training feature.

Data used to compute ADPI, along with the resulting ADPI values, are illustrated as boxplots grouped by system and training feature in Figure 8. As in the previous figure, medians are indicated by red dots, and red dotted lines connect the medians of each training modality across systems.

From the MRT of JND and ADPI subfigures, a clear trend can be observed, with the GBS showing lower values than its NGBS counterpart. The trend is not present in the percentual Weber fraction graph, which shows no variation in pitch medians and a higher value for the GBS compared to the NGBS in the duration training feature.

To determine whether there were significant differences between systems, a statistical analysis composed of a Shapiro-Wilk normality test, followed by a paired *t* test or a Wilcoxon signed rank test, was performed.

As shown in Tables 5-8, 9 out of 12 groups could not reject the null hypothesis of normality and therefore used a paired *t* test to analyze the relationship between the 2 systems.

Where normality could not be determined, the Wilcoxon signed rank test was performed instead.

According to the results, only 5 of the tests showed statistically significant differences (*P*<.05): MRT duration, PC pitch, MRT of JND duration, MRT of JND pitch, and ADPI pitch.

**Table 5.** Statistical results for the in-game key performance indicators.^a^

Tests	In-game KPIs^b^		
	MRT^c^ of JND^d^ pitch	*K* pitch	*K* duration
Shapiro-Wilk test			
*W* value	0.804	0.816	0.807
*P* value	.01^e^	.02^e^	.02^e^
Wilcoxon signed rank test			
*W* value	8	1.5	6
*P* value	.02^e^	.19	.53

a The top row of the in-game key performance indicators does not meet the normality assumption.

b KPI: key performance indicator.

c MRT: mean reaction time.

d JND: just-noticeable difference.

e Significance was defined as *P*<.05.

**Table 6.** Statistical results for normally distributed in-game key performance indicators (part 1).^a^

Tests	In-game KPIs^b^		
	MRT^c^ pitch	MRT duration	PC^d^ pitch
Shapiro-Wilk test			
*W* value	0.964	0.917	0.904
*P* value	.82	.34	.21
Paired *t* test			
*t* value (*df*)	−0.832 (10)	2.692 (9)	2.515 (10)
*P* value	.43	.03^e^	.03^e^

a The KPIs in this table met the normality assumption.

b KPI: key performance indicator.

c MRT: mean reaction time.

d PC: proportion of correct answers.

e Significance was defined as *P*<.05.

**Table 7.** Statistical results for normally distributed in-game key performance indicators (part 2).^a^

Tests	In-game KPIs^b^		
	PC^c^ duration	MRT^d^ of JND^e^ duration	ADPI^f^ pitch
Shapiro-Wilk test			
*W* value	0.972	0.909	0.926
*P* value	.91	.27	.37
Paired *t* test			
*t* value (*df*)	0.113 (9)	3.501 (9)	2.730 (10)
*P* value	.91	.007^g^	.02^g^

a The KPIs in this table met the normality assumption.

b KPI: key performance indicator.

c PC: proportion of correct answers.

d MRT: mean reaction time.

e JND: just-noticeable difference.

f ADPI: Auditory Discrimination Performance Index.

g Significance was defined as *P*<.05.

**Table 8.** Statistical results for normally distributed in-game key performance indicators (part 3).^a^

Tests	In-game KPIs^b^		
	ADPI^c^ duration	BIS^d^ pitch	BIS duration
Shapiro-Wilk test			
*W* value	0.968	0.964	0.854
*P* value	.88	.83	.07
Paired *t* test			
*t* value (*df*)	2.111 (9)	1.768 (10)	−1.294 (9)
*P* value	.06	.11	.23

a The KPIs in this table met the normality assumption.

b KPI: key performance indicator.

c ADPI: Auditory Discrimination Performance Index.

d BIS: Balanced Integration Score.

#### System Elements Comparison

**Figure 9.**
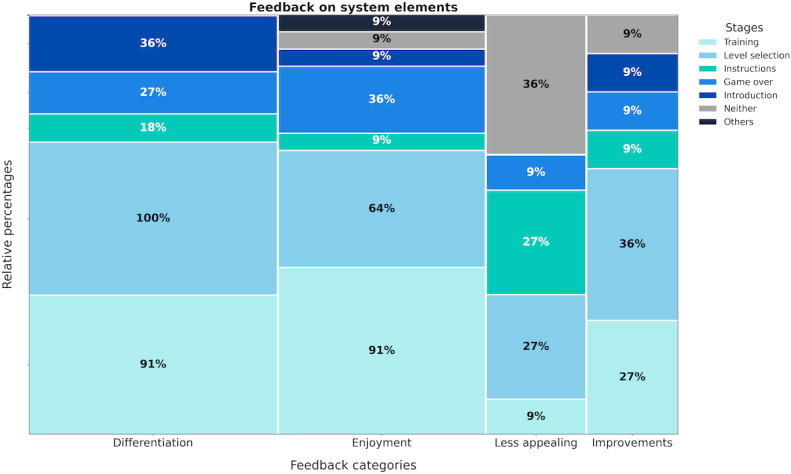
Participants’ percentual responses regarding game-based training system elements compared with the non–game-based training system in differentiation, enjoyment, less appealing, and improvements within system stages. Percentages for each individual section were calculated from the total number of participants.

At the end of the experiment, participants were asked to compare both systems and answer 4 questions, mainly about the elements of the GBS that were enhanced: differentiation, enjoyment, less appealing, and improvements compared to the NGBS, as can be observed in Figure 9. They could select as many options as they considered appropriate for each question. It was observed that *Training* and *Level Selection* were the stages of the GBS that were majorly differentiated (10/11, 91% and 11/11, 100%) and enjoyed (10/11, 91% and 7/11, 64%) by the participants compared to the NGBS. Some participants suggested smooth improvements on *Training* (3/11, 27%) for having a practical tutorial and enhancement of the reward system. *Level Selection* improvements (4/11, 36%) were suggested for clearer elements and level labels.

## Discussion

### Principal Findings

This study aimed to design a game-based auditory discrimination training system and compare it with an equivalent non–game-based version through UEQs and behavioral performance. Main findings of this work suggest that in terms of UX, the game-based system significantly improved engagement-related and hedonic dimensions while maintaining comparable auditory discrimination performance across most metrics. In contrast, the non–game-based system showed a slight advantage in perceived usability, suggesting a trade-off between simplicity and engaging elements [38]. Behavioral results showed that the non–game-based system tended toward slower but slightly more accurate responses. Despite added cognitive load, the game-based system maintained similar efficiency and no degradation of auditory discrimination, suggesting that gamification enhanced engagement without impairing performance [39].

Our study has revealed that UEQ dimensions such as attractiveness, stimulation, and novelty were consistently higher in the GBS, indicating a more engaging and appealing UX. In contrast, usability dimensions such as clarity, efficiency, and dependability showed no relevant differences between systems. These results imply that the design of game-based auditory training did not compromise usability aspects as recommended [40]. This is important since if the system is insufficiently easy to use, the user may choose to avoid the system or use it improperly even if other aspects, such as functionality, are well thought out [41], and this can drastically impact the UX and training outcomes [42]. PSSUQ results indicated no significant differences between systems in perceived usability, suggesting that both interfaces were functionally comparable. These findings suggest that the NGBS design may be more straightforward in terms of usability, which may not be the case in more interactive systems [40]. Similarly, engagement-related dimensions in the UES-SF consistently favored the GBS, particularly in terms of focused attention, aesthetic appeal, and perceived reward. Overall, these findings indicate that gamification may enhance user engagement while not differing in levels of usability.

A factor that could be relevant and influential to interpreting UX outcomes is the rate of exposure to video games. Most participants reported “low” gaming activity (<4 h/wk), indicating limited familiarity with gamified environments. Repetitive gaming experience (replay value) can influence how users perceive and interact with gamified systems [43]. Users who regularly play video games are accustomed to dynamic interfaces, reward structures, and multitasking demands, which may reduce cognitive load, in contrast with users with limited gaming experience, who may initially perceive gamified environments as more complex and less intuitive, potentially contributing to the slightly higher usability ratings observed for the non–game-based system [44]. Still, out of the 11 participants, only 2 reported higher use of video games (4‐8 h and 8-12 h), and no exploratory trend was found.

Interestingly, most of the KPIs showed nonsignificant differences between systems, with the exception of MRT (duration), MRT of JND (pitch and duration), PC (pitch), and ADPI (pitch), suggesting comparable auditory performance across systems. KPIs related to BIS showed slightly higher MRT values for the NGBS when compared to the GBS across both training features, indicating an overall slower response that could be attributed to the absence of dynamic elements in the NGBS, as supported by studies comparing tasks with and without game elements [45]. NGBS also exhibited slightly higher PC results, supported by the traditional speed-accuracy trade-off, where reaction time correlates with accuracy [46,47]. However, when comparing training features, BIS shows a clear trend: both systems exhibit positive values in the pitch feature, indicating fast and accurate performance, whereas the negative values in duration training reflect slower and inaccurate performance. Regarding MRT of JND, related to ADPI, NGBS showed longer reaction times than GBS, while Weber fraction had no variation in pitch but a larger value for GBS duration, possibly indicating lower sensibility. However, significant differences were only found for MRT of JND in both pitch and duration, as well as for ADPI pitch. Additionally, since participants in the NGBS achieved higher MRTs but lower Weber fraction values, slightly higher ADPI results were obtained. This enhances the efficiency compensation of the speed-accuracy trade-off [31].

The dual-task element introduced in the game-based system did not lead to a decline in performance, as shown by BIS and ADPI results. Conversely, participants in the GBS maintained similar BIS values to the NGBS and even showed an improvement in ADPI. Similar results were found in a study involving an auditory discrimination gamified system [48], where the enhancement of learning was attributed to the reward signals playing a reinforcement role. In the present study, this may suggest that the different aspects of the game-based system (eg, animated success/error, points, lives, visual stimuli, background music) served to mitigate the monotony of the primary task—repetitive auditory discrimination—rather than acting as a source of distraction, thereby enhancing engagement and maintaining performance despite the additional cognitive load [49]. This finding also highlights the potential benefits of the infinite runner game genre in these tasks, suggesting recommended levels of cognitive load to mediate task difficulty and user performance [50].

Studying the relationships between engagement and outcomes has been suggested in game-assisted learning, as motivation may play a crucial role in effectiveness [51]. This is supported by other studies that suggest that user-perceived experience and cognitive load may be good predictors of cognitive training performance [52]. Thus, players’ interactions with the systems’ mechanics (dynamics) and aesthetics (feelings of players during those interactions) [53] may enhance user-perceived experience while balancing cognitive load to optimize performance.

This study has several limitations that should be considered. First, the sample size was modestly small and limited to young adults. This may bias behavioral performance significance and restrict the generalizability of the findings to other populations but may serve as a pilot study for treating auditory processing disorders [54] or hearing device users [55]. The sample size is considered enough for addressing usability-related measurements; however, the significance of differences in behavioral performance should be interpreted with caution. Second, the sample presented a slightly unbalanced gender distribution with a male-to-female ratio of 1.75:1. Although acceptable for initial studies, it can introduce bias in UX perceptions. Future studies should aim for a more balanced sample in gender distribution to ensure broader generalizability of the findings. Moreover, the evaluation focused on short-term performance and UX and therefore does not allow conclusions about long-term training effects, as auditory processing is subject to different time courses of plasticity [56]. Although the systems were carefully controlled to isolate the effect of gamification, other factors such as individual familiarity with video games and personal preferences may have influenced engagement outcomes [57].

The usability-engagement tradeoff seen in the results between both systems could be addressed through adaptive, user-centered interface design. Interface complexity may adapt to user performance and familiarity, offering simpler layouts for novices and more dynamic environments for experienced users [58]. Additionally, progressive disclosure can introduce game mechanics, scoring, and feedback incrementally, maintaining clarity while leveraging motivational benefits [59]. Over time, neuroplasticity-related adaptation may further enhance usability, as repeated exposure to gamified environments improves users’ efficiency and perception of the interface [49]. Consequently, although non–game-based systems may provide immediate usability advantages, game-based systems could match or surpass them as familiarity increases and cognitive load decreases [59].

Taken together, the present findings highlight the value of incorporating gamification into auditory discrimination training, particularly as a strategy to enhance engagement and sustained attention without negatively affecting performance [52]. A key contribution of this study lies in directly comparing game-based and non–game-based systems under controlled conditions, which offers a more nuanced perspective on how specific game elements may influence UX and behavioral outcomes, although game elements may vary across conditions and other outcomes [60]. From an applied standpoint, the observed improvements in engagement-related domains suggest that such approaches may support greater adherence to auditory training protocols, which is critical for compliance in the real-world aural rehabilitation process [61]. Future research should further explore these effects over longer periods to determine the systems’ efficiency in longitudinal studies, as it is suggested that gamification may perform more efficiently compared to control groups [62].

### Conclusion

This study compared the use of gamification in auditory discrimination tasks. The introduction of gamified dual tasks favors a better qualitative perception of UX, particularly those related to engagement- and attention-related domains but may complicate interface understanding in a first approach, since more elements are displayed that require higher attention and cognitive workload when starting with system interaction. This is supported by qualitative feedback items suggesting longer tutorials. The introduction of a dual task did not significantly affect performance in auditory discrimination tasks for duration and pitch features in the GBS compared to the NGBS, as shown by BIS and ADPI, even presenting slightly better scores for the GBS, although not significant except for ADPI in pitch. This also suggests that gamified discrimination tasks tend to decrease reaction time as part of the game mechanics, yielding focused attention and faster responses.

The current research also analyzes some key gamification elements that impact user perception. Findings suggest that the infinite runner game genre is flexible with auditory tasks and enhances engagement and attentional resources, supported by both questionnaire and discrimination performance results, providing important insights into genre selection for training compatibility. Additionally, longer exposure time is something to consider, as only one training session was evaluated and could yield slower responses due to first impressions. This may guide further research on the long-term impact of gamification on user evaluation and performance in auditory tasks, where improvements can be measured to compare both systems based on users’ responses. More research is still needed in game-based training and user-centered design for auditory technology development to deliver better experiences to youths and children in aural habilitation and rehabilitation processes that enhance attention, fun, and lead to better auditory discrimination. Overall, our findings support the use of gamified, dual-task paradigms as a promising strategy to enhance UX and engagement in auditory discrimination training without compromising short-term performance. Future longitudinal studies should examine whether these experiential benefits translate into sustained improvements in auditory performance and clinical outcomes.

## Supplementary material

10.2196/92496 Multimedia Appendix 1 Differences in game-based training and non–game-based training features.

## Abbreviations

ADPI: Auditory Discrimination Performance Index
BIS: Balanced Integration Score
GBS: game-based training system
JND: just-noticeable difference
KPI: key performance indicator
MRT: mean reaction time
NGBS: non–game-based training system
PC: proportion of correct answers
PSSUQ: Post-Study System Usability Questionnaire
UEQ: User Experience Questionnaire
UES-SF: User Engagement Scale-Short Form
UX: user experience
