# Supplementary material for: Tonal Auditory Discrimination Training in Youths: Design, Implementation, and Evaluation of Game-Based Versus Non–Game-Based Systems in a Cross-Sectional Study
Source: JMIR Serious Games. 2026 Jun 22;14:e92496. doi: 10.2196/92496 (PMC13286528; doi:10.2196/92496)
Supplement: Multimedia Appendix 1 [file games-v14-e92496-s001.docx]

# Appendix

Table S1. Differences in Game-Based Training and Non-Game-Based Training Features.

| **Feature (Description)** | **Non-Game** | **Game** |
| --- | --- | --- |
| Logos presentation | - AIR and NeuroTechs logos are been shown | - AIR and NeuroTechs logos are been shown |
| Game Cover | - Presentation of project - *Non-commercialization* legend - Acknowledgements | - **Intro Music (8-bit based)** - **Dynamic visuals** - Presentation of project - *Non-commercialization* legend - Acknowledgements |
| Game Menu (Selection) | - Button for level selection - Options for pitch, intensity, duration and position - Training remaining time | - **Bridge Music (8-bit based)** - **Dynamic visuals** - **Map** for level selection - Options for pitch, intensity, duration and position - Training remaining time |
| Instructions | - Sound level adjustment - Cue-target auditory discrimination task instruction - Interaction commands to use - Time objective (achievements) | - **Pop-Up GUI** - Sound level adjustment - Cue-target auditory discrimination task instruction - Interaction commands to use - Time objective (achievement) - **Score points objective (achievement)** |
| Training screen | - Feature training display - Training remaining time display - Attempts - Success and error display | - Feature training display - Training remaining time display - **Lives (hearts)** - **Start sound** - Success and error **sounds** - Success and error **animations** - **Game task score points generation** - **Pause Button** |
| Level-end screen | - Level end screen when time or attempts are over - Back to Menu Option | - Level **Success Screen** when time is over - Level **Game Over** when lives are over - Back to Menu and **Restart Level** Option |
| Training session-end screen | - Time limit achieved - Day training end - Questionnaire reminder - Questionnaire screen | - Time limit achieved - Day training end - Questionnaire reminder - Questionnaire pop-up |
| Saving Data | - Training time saving - Training feature saving - Intervals, answer and time saving - Questionnaire answers saving | - Training time saving - Training feature saving - Intervals, answer and time saving - **Score points saving** - Questionnaire answers saving |
